# Frequency, Severity and Ergonomic Awareness of Work-Related Musculoskeletal Disorders Among Australian Optometrists: A Cross-Sectional Survey

**DOI:** 10.1007/s44402-026-00118-z

**Published:** 2026-06-16

**Authors:** Enoch A. Appathurai, Kwang M. Cham, Peter V. S. Lee, Bang V. Bui, Nilushi Kodikarage, Dylan Loh, Duy Ho, James Burt, Joshua Goundar, Selwyn M. Prea

**Affiliations:** 1 Department of Medicine, The University of Melbourne, Parkville, Victoria Australia; 2 Department of Optometry and Vision Sciences, The University of Melbourne, Parkville, Victoria Australia; 3 Department of Biomedical Engineering, The University of Melbourne, Parkville, Victoria Australia

**Keywords:** Ergonomics, Musculoskeletal injury, Occupational hazards, Optometrists, Workplace safety.

## Abstract

**Purpose:**

Work-related musculoskeletal disorders (WMSDs) are highly prevalent among healthcare professionals, including optometrists. This study aimed to assess the frequency, severity and contributing factors of WMSDs among Australian optometrists, as well as evaluate their awareness and implementation of ergonomic practices.

**Methods:**

A cross-sectional online survey was distributed to optometrists in Victoria and South Australia. The survey collected data on demographics, work characteristics, ergonomic awareness, frequency and severity of musculoskeletal discomfort and contributing factors. Quantitative data were analysed using descriptive statistics and correlation analysis. Free-text responses were examined using inductive thematic analysis.

**Results:**

Of 119 respondents, 84% reported neck discomfort, 71% shoulder discomfort and 73% lower back discomfort. Pain severity was highest in the neck and shoulders (mean ~6.5/10). Strong Pearson correlations were found between neck, shoulder and upper back discomfort. Repetitive tasks, static postures and awkward positioning were identified as primary contributors to discomfort. Slit lamp-based tasks were most frequently associated with exacerbating pain. Only 20% of respondents were aware of workplace health and safety policies and 97% had not received employer-led training on injury reduction. Thematic analysis revealed significant impacts on professional performance and personal wellbeing.

**Conclusions:**

WMSDs are highly prevalent among Australian optometrists, particularly affecting the upper body. There is a lack of ergonomics education and workplace support. These findings highlight the need for improved ergonomics training in optometry education, implementation of workplace health and safety protocols and investment in ergonomic equipment. Future interventions should focus on addressing the identified risk factors and promoting a culture of safety in optometric practice.

Key Points

- Work-related musculoskeletal disorders are highly prevalent among practising optometrists, particularly persistent neck, shoulder and upper back pain, strongly linked to repetitive tasks and static postures.
- Ergonomics education and employer-led safety training are rare, and most clinics lack formal workplace health and safety policies, leaving clinicians without structured support to reduce injury risk.
- Integrating ergonomics teaching, employer-supported risk assessments and investment in adjustable equipment can reduce clinician pain, support workforce longevity and protect continuity and quality of patient care.

## Introduction

Work-related musculoskeletal disorders (WMSDs) are highly prevalent (>75%) in healthcare professionals [1]. WMSDs encompass a range of conditions affecting the muscles, nerves, tendons, joints, cartilage and spinal discs, often resulting from sudden exertion, repetitive motions or sustained postures [2]. Despite awareness of ergonomic practices, the prevalence of WMSDs in healthcare professionals remains significant, leading to discomfort, reduced productivity and increased healthcare costs [3]. This study aims to consider the frequency and risk factors for WMSDs among Australian optometrists through a survey. Building on the work of Long et al. [4], this study seeks to provide detailed insights and actionable recommendations to improve occupational health and ergonomics within the profession.

Optometrists face unique ergonomic challenges due to the physical demands of their work, which often involves constrained postures and repetitive tasks [4]. Past studies have reported that 82% of Australian optometrists reported experiencing WMSDs, with the most common findings being in the neck, shoulder and lower back [5]. Similar findings have also been reported among optometrists in Canada and the United Kingdom [6, 7]. WMSDs are a point of concern for the optometry profession as they pose a significant threat to the longevity of the workforce [5, 8]. Optometrists who started their tertiary qualification directly after high school may work more than 40 years in the profession [8]. This presents a long period during which repetitive tasks can exacerbate discomfort. Severe or untreated conditions may force optometrists to reduce their workload or even retire early, leading to a loss of experienced practitioners in the field. Additionally, WMSDs have been associated with increased stress, leading to poorer wellbeing in optometrists [9].

The current understanding of WMSDs within the Australian optometry field is largely informed by work conducted by Long et al. [5, 8]. These studies involved a multistage investigation of WMSDs through a survey (initially conducted in 2008) and subsequent interviews and on-site examinations of work procedures (2012). Since then, there have not been any relevant follow-up studies. In the intervening period, the Australian workforce has grown substantially, increasing by over 32% between 2011 and 2019 alone [10]. The practice environment has also evolved, with the broader adoption of digital instrumentation and shifting models of care, which may have altered the ergonomic demands experienced by contemporary optometrists. Therefore, updated data are needed to determine whether the WMSD burden has changed and to inform ergonomic strategies that reflect the current workforce.

Furthermore, there were several limitations to Long’s studies on WMSDs. The survey did not collect data on height, weight or body mass index (BMI—a measure involving the height and weight of participants). Ghazarian and Bither [11] highlighted variations in WMSD risk attributable to gender and body size, while Domaradzki and Koźlenia [12] identified BMI as a predictor of WMSD risk. Further work by Long et al. [8] identified various characteristics influencing the risk of WMSDs, such as control over the environment, which were not considered initially. Ergonomics training is crucial in reducing the prevalence of WMSDs, as evidenced by studies such as Kaup et al. [13] and Mewada and Shukla [14]. However, the survey did not assess the awareness of posture and ergonomics among optometrists, nor did it evaluate their understanding of relevant policies, procedures or guidelines. There was limited evaluation of the impact of WMSDs on daily living, both in work and personal activities. Dhimitri et al. [15] reported that more severe discomfort can affect work performance significantly, impact the healthcare sector and increase absenteeism. Diaconita et al. [6] reported that tasks requiring prolonged static postures are major contributors to WMSDs. On the other hand, Fethke et al. [16] stated that a more detailed analysis of task-specific risk factors will aid in developing effective ergonomic interventions. Furthermore, there has been limited evaluation of the modifications made to address these risk factors, as well as no assessment of the effectiveness of these interventions.

Building on the identified limitations of prior research, including the absence of ergonomics awareness data, limited assessment of daily living impacts and lack of follow-up since 2012, the primary objective of this study is to describe the self-reported frequency, severity and impact of WMSDs among optometrists in Australia using a contemporary sample. The secondary objective was to identify specific clinical tasks contributing to WMSDs and to assess the awareness, training and implementation of ergonomic practices within the profession.

## Methods

### Survey Development

The survey was developed iteratively by three authors (EAA, SMP and KMC) with feedback from colleagues who were clinicians or educators in the field of optometry to ensure its comprehensiveness and relevance, consistent with recommended approaches to content validation in health measurement [17]. The survey was developed de novo, as no single existing validated instrument addressed fully the combined focus on WMSD frequency, ergonomic awareness and the occupational context of optometric practice. Survey items were informed by the structure and body-region framework of the Standardised Nordic Musculoskeletal Questionnaire [18], a widely used and validated tool in occupational musculoskeletal research, but were not adapted directly from it. Content validity was established through iterative review by clinician and educator colleagues. This iterative process involved assessing various focuses such as demographics, professional activities, ergonomic awareness, frequency of WMSDs and the impacts and responses to WMSDs. A pilot survey was conducted initially with 13 participants sourced from the Victorian optometry community, with preliminary data examined and feedback gathered from participants. Following the pilot, several revisions were made: ambiguous question wording was clarified (particularly in items relating to posture frequency and ergonomic training), and two redundant items were removed. This paper outlines the results of the finalised survey (see Supplementary Material).

### Survey Content

#### Demographics and Professional Information

To understand the background and work context of the participants, this survey collected detailed demographic and professional information. This section included questions on gender, age, height, weight, dominant hand, qualification location and years of practice. Additionally, it gathered data on weekly working hours and the number of patients seen daily, as well as specific tasks performed, such as eye examinations, frame selection and dispensing. This approach addressed previously identified gaps by including critical demographic factors such as height, weight and BMI, which have been linked with WMSD risk [13]. Collecting information on qualification location helped to understand the influence of training environments on ergonomic practices.

#### Ergonomic Awareness and Practices

This section aimed to assess participants’ awareness of ergonomic practices and their implementation in the workplace. Questions addressed the awareness and self-assessment of posture, availability of workplace health and safety (WHS) protocols and training received on ergonomic practices and risk assessments at the workplace. Participants were also asked about their control over work pace and input on equipment selection. By evaluating these aspects, the survey contributed to existing gaps regarding the awareness and training in ergonomics [14, 15]. It also explored how well these practices are being taught and implemented.

#### Musculoskeletal Discomfort

To evaluate the frequency and severity of WMSDs, this section included questions on the discomfort experienced in various body regions such as the neck, shoulders, upper back and lower back. It also covered the severity and duration of discomfort, specific work-related activities contributing to the discomfort and any modifications made to alleviate it. Additionally, the survey inquired about health interventions, including medication use and consultations with healthcare professionals, as well as the impact of WMSDs on health and daily living activities. This comprehensive approach meets the gaps identified in understanding the severity of discomfort and the effectiveness of interventions. It allows for a thorough assessment of how WMSDs impact daily living and professional activities, which is essential for prioritising interventions and allocating resources effectively [16, 19].

#### Survey Distribution

The online survey was conducted on the Qualtrics survey management software (qualtrics.com) and distributed to a convenience sample of optometry colleagues in Victoria and South Australia, predominantly through an email newsletter advertisement by the Optometry Victoria and South Australia Association. As participation was voluntary and the survey was distributed through a single professional association network, a convenience sample was obtained. This approach, while practical for an exploratory study of this kind, meant that findings should be interpreted with respect to this respondent group and may not be fully representative of all Australian optometrists across different states, practice models or employment settings. Informed consent was obtained, and participation in the study was voluntary. This study was approved by the University of Melbourne Human Research Ethics Advisory Committee (ID: 28636).

#### Survey Analysis

Qualtrics data was exported to Microsoft Excel (microsoft.com) and GraphPad Prism 6.0 (graphpad.com) for analysis. Incomplete survey responses were excluded from the analysis. Summary variables were summarised with means, standard deviations, frequencies and percentages. Correlation analysis was performed to identify any significant associations for the occurrence of WMSDs in eight body regions (neck, shoulders, upper back, lower back, knees, ankles/feet, wrist/hands and hips/thighs). For binary variables (presence/absence of discomfort), Pearson correlation coefficients were used (equivalent to the phi coefficient, for dichotomous data and thus not requiring assumptions of normality). For pain severity scores (0–10 scale), Spearman’s rank correlation coefficients were used, as these data are ordinal and may not satisfy assumptions of normality or linearity required for Pearson correlation. Spearman correlation does not assume normality and assesses monotonic relationships based on ranked data. A Bonferroni correction was applied to account for multiple comparisons, with statistical significance set at *p* < 0.0018 (*α* = 0.05/28 = 0.0018). No multivariable analyses were performed; therefore, findings represent unadjusted associations. Inductive thematic analysis was performed to analyse free-text comments by five authors (NK, DL, DH, JB and JG) [19, 20], which was checked and verified by two supervising authors (SMP and KMC). Collectively, all authors reached consensus on the final overarching themes and sub-themes.

## Results

### Demographics and Participant Information

Participant demographics and workplace characteristics are summarised in Table 1. Of 162 responses, 119 participants provided consent and completed the survey. The sample was predominantly female and early-to-mid career, broadly consistent with the national optometry workforce as reported by the Optometry Board of Australia’s Registration dataset (as of September 2024) [21]. Most participants worked full-time hours and conducted between 11 and 15 patient consultations per day (Table 1), with eye examinations comprising the dominant clinical activity. A smaller proportion of time was allocated to administration, dispensing and frame selection, while 7% reported involvement in other activities, including clinical teaching and post-operative reviews.

**Table 1:** Participant demographics and work characteristics (*n* = 119).

Category	Variable	*n* (%)/Mean ± SD
Demographics	Right-handed	112 (94%)
	Left-handed	7 (6%)
	Qualified in Australia/New Zealand	117 (98%)
	Qualified elsewhere	2 (2%)
	Height (cm)—all	168 ± 10
	Height (cm)—male/female	180 ± 8/164 ± 7
	Weight (kg)—all	69 ± 15
	Weight (kg)—male/female	80 ± 13/65 ± 13
Years of experience	<5 years	34 (29%)
	5–10 years	26 (22%)
	10–15 years	15 (13%)
	>15 years	44 (37%)
Weekly workload	<10 h	11 (9%)
	10–19 h	10 (8%)
	20–29 h	13 (11%)
	30–39 h	57 (48%)
	40+ h	28 (24%)
Daily patient load	0–5 patients	8 (7%)
	6–10 patients	33 (28%)
	11–15 patients	54 (45%)
	16+ patients	24 (20%)
Main tasks performed	Eye examinations	112 (94%)—28 ± 10 h/week
	Administration	80 (67%)—6 ± 8 h/week
	Dispensing/Repairs	41 (34%)—2 ± 2 h/week
	Frame selection	30 (25%)—3 ± 3 h/week
	Other tasks	8 (7%)

*SD* standard deviation.

Data on height and weight were collected to allow for a more comprehensive analysis of risk factors. The average height across all participants was 168 cm ± 10 (male = 180 cm ± 8, female = 164 cm ± 7), and the average weight was 69 kg ± 15 (male = 80 kg ± 13, female = 65 kg ± 13).

### Prior Understanding of Ergonomics and Reducing WMSDs

Table 2 summarises participants’ prior understanding of ergonomics and implementation of strategies to reduce WMSDs. A notable disconnect was observed between posture awareness and perceived effectiveness: while most respondents reported being often or always aware of their posture, the majority rated their actual posture as only average or below average (Table 2). Formal workplace safety infrastructure was largely absent, with very few respondents confirming that WHS policies, risk assessments or employer-led training were in place at their workplace (Table 2).

**Table 2:** Summary of prior understanding of ergonomics and reducing work-related musculoskeletal disorders (WMSDs).

Category	Item	*n* (%)
Posture awareness and rating	Always aware of posture	15 (14%)
	Often aware of posture	47 (45%)
	Rated posture as average	46 (44%)
	Rated posture as below average or poor	44 (42%)
Workplace safety and ergonomics	Workplace has WHS policies	21 (20%)
	Risk assessment conducted	2 (2%)
	Employer training on injury reduction	3 (3%)
	Employer training on ergonomic setup	5 (5%)
Awareness of formal guidelines	Aware of legislation/guidelines (Agree or Strongly Agree)	9 (9%)
	Not aware (Disagree or Strongly Disagree)	70 (67%)
Workplace autonomy	Control over setup (Agree or Strongly Agree)	28 (27%)
	Control over work pace (Agree or Strongly Agree)	38 (36%)
Formal education and CPD	Received training for injury prevention in optometry	11 (11%)
	Received training for ergonomic setup	14 (13%)
	Completed CPD in ergonomics	4 (4%)

*CPD* continuing professional development, *WHS* workplace health and safety.

Awareness of relevant legislation and formal guidelines was similarly low, and most respondents reported limited control over their clinical environment and work pace (Table 2). Ergonomics education during optometry training was rare, as was participation in continuing professional development in this area (Table 2).

### Frequency and Severity of Discomfort

With regards to the location of discomfort, the neck and shoulders were the most frequently affected regions, affecting 84 and 71% of respondents, respectively. These areas also recorded the highest mean pain severity scores (~6.5 out of 10), suggesting a considerable burden of symptoms. Discomfort in the upper back (65%) and lower back (73%) was also common, albeit less so, with mean pain scores of 5.9 and 6.0, respectively.

Pain in the neck, shoulders and upper back appears to be part of a cluster of interrelated anatomical regions that exhibit high co-reporting and co-severity of discomfort. This was evidenced by strong correlations between neck and upper back discomfort (*r* = 0.47, *p* < 0.0018; Table 3), shoulder and upper back discomfort (*r* = 0.46, *p* < 0.0018; Table 3) and neck and shoulder discomfort (*r* = 0.37, *p* < 0.0018; Table 3). The correlation was even stronger for pain severity scores, with neck-shoulder pain severity correlation at *r*_s_ = 0.74, *p* < 0.0018 (Table 4) and both neck-upper back (*r*_s_ = 0.64, *p* < 0.0018) and shoulder-upper back (*r*_s_ = 0.65, *p* < 0.0018) correlations (Table 4). All these associations remained statistically significant following Bonferroni correction. In contrast, discomfort in the lower back showed weaker correlations with other regions.

**Table 3:** Discomfort correlations for *n* = 119 optometrists.

	Neck	Shoulder	Upper back	Lower back	Elbow	Wrist/Hand	Knee	Ankle/Foot
Neck	1.0							
Shoulder	0.37*	1.0						
Upper back	0.47*	0.47*	1.0					
Lower back	0.10	0.04	0.10	1.0				
Elbow	−0.07	0.17	0.05	0.12	1.0			
Wrist/Hand	0.10	0.05	0.18	0.04	0.06	1.0		
Knee	0.09	0.18	0.12	0.20	0.08	0.05	1.0	
Ankle/Foot	−0.01	0.09	0.12	0.22	0.34*	0.22	0.40*	1.0

Values represent Pearson correlation coefficients (*r*) for co-occurrence of reported discomfort between body regions (*n* = 119). Bonferroni correction was applied for multiple comparisons (*α* = 0.05/28 = 0.0018).

* indicates statistically significant correlations after correction.

**Table 4:** Pain severity correlation for 100 optometrists.

	Neck	Shoulder	Upper back	Lower back	Elbow	Wrist/Hand	Knee	Ankle/Foot
Neck	1.0							
Shoulder	0.74*	1.0						
Upper back	0.64*	0.65*	1.0					
Lower back	0.16	0.11	0.18	1.0				
Elbow	0.01	0.16	0.03	−0.01	1.0			
Wrist/Hand	0.08	0.06	0.14	0.04	0.09	1.0		
Knee	0.25	0.23	0.16	0.02	0.07	0.05	1.0	
Ankle/Foot	0.00	0.10	0.03	0.06	0.39*	0.28	0.38*	1.0

Values represent Spearman’s rank correlation coefficients (*r*_s_) for associations between discomfort severity scores across body regions (*n* = 100). Bonferroni correction was applied for multiple comparisons (*α* = 0.05/28 = 0.0018).

* indicates statistically significant correlations after correction.

Contextual data about the timing and persistence of discomfort further reinforce its chronic and work-related nature. Most respondents reported that neck, shoulder and back discomfort occurred both during work and persisted for hours afterwards, with 61% reporting lingering neck discomfort. Notably, a significant portion of respondents indicated that pain had persisted for more than 5 years, particularly for the upper back (54%), neck and shoulders (both >40%), highlighting the long-term impact of these issues and cumulative strain from years in clinical practice. Interestingly, wrist and hand discomfort showed a different pattern: over half of the participants (52%) reported that pain occurred only while working, suggesting a task-specific component such as instrument handling, keyboard use or fine motor demands.

### Contributing Factors to Discomfort

Participants identified several contributing factors to their physical discomfort, with repetitive tasks and postural strain emerging as key drivers across multiple body regions. Repetition was reported as the primary contributor to discomfort in the elbow (79%), neck (76%) and wrist/hand (59%). Static posture was strongly associated with pain in the neck (71%), lower back (57%) and knees (52%), reflecting the physical demands of prolonged seated and fixed-position tasks common in optometric practice. Awkward or cramped positions were frequently cited as aggravating discomfort in the neck (68%), upper back (60%) and ankle/foot (39%). In particular, bending and twisting movements were noted as contributors to lower back pain (53%) and neck pain (59%). Shoulder discomfort was linked to both repetition (64%) and awkward posture (58%).

When asked about specific clinical activities that exacerbated discomfort, slit lamp-based tasks, including fundoscopy and gonioscopy, were commonly reported. These procedures were reported to worsen pain in the neck (82%), shoulders (60%), upper back (60%) and wrist/hand (43%). Computer use and writing were also stated, especially for the neck (60%), lower back (55%), upper back (50%) and wrist/hand (48%). Using the phoropter contributed to shoulder (49%), neck (41%) and upper back (39%) discomfort. Trial frame and handheld tools, while less frequently cited, still had notable effects, primarily on the shoulders (30%) and lower back (27%).

### Thematic Analysis

WMSDs had significant repercussions across both professional and personal domains of optometric practice (Table 5). Many optometrists reported chronic pain that affected their clinical performance and career trajectory. Professional consequences included reduced working hours, early retirement or complete career change. Some participants quoted ‘feeling depressed and deflated’ due to persistent chronic discomfort, with consultations often having to be adapted to manage the pain.

**Table 5:** Overarching themes and sub-themes associated with work-related musculoskeletal disorders (WMSDs) in optometric practice.

Theme	Sub-theme	Survey comments
Impact of WMSDs	Professional consequences	‘…From final year of university I have experienced back pain and it has almost pushed me to leave the profession but I love the other parts of the job too much’.
	Personal consequences	‘I have suffered for many years with work related upper back/neck/shoulder pain which caused me much discomfort, particularly in the last year. This work-related issue impacted my home life and everyday living activities’.
Self-coping mechanism	Seeking medical treatments and exercise	‘I see a myotherapist and physiotherapist who have provided me with stretches and exercises to strengthen my upper back and shoulders to prevent further injury after a current injury/issue caused by positioning at work’
	Adjustments to workplace setting	‘The digital phoropter I have used for the last 15 years is way more comfortable on the shoulders/neck compared to the manual phoropters I always used before that, so the console can sit on the desk in front of me rather than having to reach up to the phoropter’.
Limited understanding of workplace ergonomics	Gap in knowledge preventing risk-mitigating behaviour	‘I don’t feel that this is given enough importance in the realm of optometric practice! And often comes as a surprise when you begin working as a new graduate and realise the physical demands of the job. I feel this should be addressed in university courses as a mandatory part of teaching clinical skills!’
	Gap in training for risk management in the workplace	‘We need to make it mandatory for all optometrists to have Occupational Health and Safety come in to assess the workplace... things like fixing chairs and tables to make it a good height often gets overlooked’.
Unmet employee expectations	Employee expectations	‘Would be good to have more awareness and solutions provided within the community. A lot of concerns are brushed off and accepted as part of the profession. Would also be good if ergonomics is considered when designing setup of consult rooms...’

WMSDs had a substantial impact on the quality of life and mental wellbeing of some individuals. Respondents reported difficulties with daily activities, such as lifting their children or playing recreational sports, due to chronic work-related pain. Financial strain from ongoing physiotherapy or other treatments, often not reimbursed, further compounded the issue.

To manage discomfort, most optometrists adopted self-directed coping mechanisms. Physiotherapy, massage therapy and exercise programmes such as Pilates were commonly undertaken, even though relief was only temporary.

Some participants relied on analgesics, but with limited benefit. Some invested in their own ergonomic equipment, such as chairs or stand-up desks. Digital phoropters also helped reduce awkward postures and improved physical comfort during consultations.

There was strong agreement that workplace ergonomics is inadequately addressed during optometric education, with most reporting only a single lecture on the topic. Many clinicians also lacked control over the clinical room setup, hence constraining their ability to implement ergonomic improvements. Respondents suggested that this knowledge gap should be addressed through more robust clinical teaching and post-graduation support.

Moreover, ergonomics training was often patient-focused, neglecting the needs of clinicians. Participants advocated for mandatory occupational health and safety assessments in optometric settings and voiced disappointment in employer responses, with concerns frequently dismissed. Many felt ergonomic challenges were treated as ‘part of the job’ rather than risks to be mitigated. Respondents proposed that investing in ergonomic equipment, allowing more frequent breaks and offering mental health or rostered days off will better support clinician wellbeing.

## Discussion

The demographic profile of respondents aligned closely with the national optometry workforce, with the sample predominantly female and early-to-mid career, suggesting reasonable representativeness within the Victorian and South Australian context. The inclusion of height and weight data offers a novel contribution to the optometry ergonomics literature, providing a foundation for future workstation design and equipment placement considerations specific to this population.

Patterns of clinical workload provide important context for understanding musculoskeletal risk exposure. Most respondents worked full-time or extended hours and conducted high volumes of patient consultations predominantly centred on eye examinations (Table 1). This sustained, repetitive workload, combined with the administrative and dispensing duties that occupy a meaningful proportion of practice time, creates cumulative musculoskeletal load across multiple body regions and task types, reinforcing the case for ergonomics training and workplace design tailored to realistic workload patterns.

A consistent pattern of inadequate ergonomic infrastructure was evident across workplace, educational and policy domains. Despite most respondents reporting frequent awareness of their posture, the majority rated their actual posture as only average or below average; a discrepancy consistent with prior evidence that health professionals often recognise workplace strain but lack the tools or environment to act on it effectively [22]. Formal workplace safety structures were largely absent, with very few respondents confirming that WHS policies, risk assessments or employer-led training were in place at their workplaces (Table 2). These findings suggest that WMSD prevention is not integrated routinely into optometric workplace practices and that structured onboarding and occupational health policies specific to clinical optometry settings are warranted. A likely contributing factor to this pattern is the limited integration of ergonomics into both optometry curricula and continuing professional development, with very few respondents recalling any formal training in injury prevention or ergonomic setup during their education or post-graduation (Table 2). This systemic gap may leave optometrists ill-prepared to recognise or manage musculoskeletal risk across the course of their careers. Compounding this, most respondents reported limited control over their clinical environment and work pace, and very few were familiar with relevant WHS legislation (Table 2), suggesting that even those motivated to work more safely may lack both the autonomy and the foundational knowledge to do so.

**Fig. 1:**
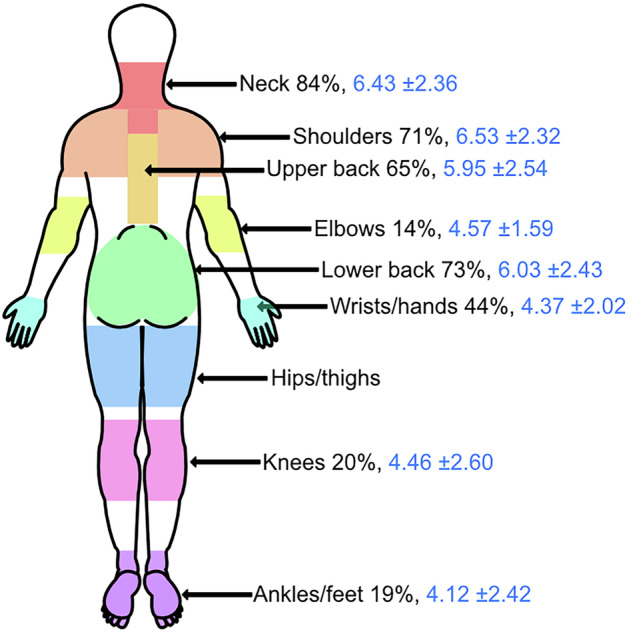
Discomfort by body region experienced by *n* = 100 optometrists. Blue data represents pain severity score (out of 10) ± SD. SD standard deviation.

Upper body musculoskeletal discomfort represented the greatest burden in this sample, both in frequency and severity (Fig. 1), consistent with prior research attributing this pattern to sustained visual focus, repetitive tasks and prolonged static postures inherent to clinical optometry [3, 21].

The clear anatomical clustering of symptoms across the neck, shoulders and upper back, supported by strong co-reporting and severity correlations (Tables 3 and 4), suggests a shared ergonomic origin, likely stemming from poor workstation setup, inadequate posture during eye examinations and/or the use of non-adjustable equipment. These regions appear to function as a kinetic chain under biomechanical stress, reinforcing the value of ergonomic interventions that address the entire upper body rather than targeting isolated joints.

While lower back discomfort was moderately prevalent, it showed weaker correlations with upper body pain, indicating that it may arise from more isolated ergonomic or structural factors, such as prolonged unsupported sitting or poor lumbar support, rather than being a part of the upper body postural cluster. This distinction is important when designing workplace modifications, as lower back pain may require different ergonomic solutions (e.g., chair adjustments, lumbar cushioning) than neck and shoulder discomfort.

The findings from this study reveal that the most significant contributors to musculoskeletal discomfort among optometrists are repetitive tasks, static posture and awkward positioning, particularly affecting the upper body. Repetitive motion was the leading reported cause of pain in the neck, while static posture was closely linked with neck and lower back discomfort. This supports existing literature highlighting the burden of a sustained seated position and fixed gaze during clinical procedures [21]. Awkward or cramped positions were particularly problematic for the upper back and neck, while bending/twisting movements were implicated in lower back and neck strain. These patterns reinforce the idea that both static load and constrained movement contribute significantly to physical stress in clinical practice.

In addition to these general contributors, specific optometric tasks were identified as key aggravators of discomfort. Procedures requiring prolonged concentration and positioning at the slit lamp, such as fundoscopy and gonioscopy, were consistently linked with pain in the neck, shoulders and upper back. Interestingly, even seemingly minor activities like trial frame use and handheld testing tools had a measurable impact on shoulder and lower back discomfort. These results suggest that ergonomic modifications to slit lamp stations, seating and work posture, combined with rest breaks and task variation, may assist in reducing long-term musculoskeletal burden among optometrists.

Qualitative findings from the free-text responses reinforced and extended the quantitative results, illustrating the breadth of impact that WMSDs can have on both professional and personal domains. Chronic discomfort was associated with professional adaptations, including modified consultation posture and reduced clinical hours, reflecting the degree to which pain had affected routine practice. Beyond the clinic, respondents described discomfort interfering with daily activities and family responsibilities, consistent with evidence that occupational musculoskeletal conditions extend their effects well beyond the workplace [3]. The financial costs of self-funded treatment compound the burden for some individuals.

The widespread use of self-directed coping strategies, including physiotherapy, massage and personal investment in ergonomic equipment, highlights the degree to which optometrists are managing musculoskeletal risk largely without formal workplace support. That respondents frequently reported an inability to influence the physical layout of their consulting rooms suggests that structural and organisational constraints may limit the effectiveness of individually motivated ergonomic behaviour, even where the motivation exists. These findings are consistent with the broader literature indicating that individual-level interventions are insufficient without complementary organisational and environmental change [22].

Taken together, the qualitative data point to a professional culture in which musculoskeletal discomfort has come to be accepted as an inherent feature of optometric practice rather than a modifiable occupational risk. This cultural normalisation, combined with the limited ergonomics education identified in the quantitative findings, may help explain why WMSD rates have remained high over time. These observations suggest that there may be meaningful opportunities to strengthen ergonomics integration in optometric education and to foster greater awareness of workplace health responsibilities among employers and professional bodies, though prospective and interventional research will be needed to determine which approaches are most effective.

While this study offers valuable insights into the frequency of WMSDs among Australian optometrists and highlights clear opportunities for prevention, there are several considerations to be acknowledged. The cross-sectional survey design was useful in capturing the current practices and experiences of clinicians. However, it does not allow for causal relationships to be established with certainty. The study gathered data from a convenience sample of optometrists in Victoria and South Australia, largely from a single professional network, and so the findings represent this respondent group and may not be reflective of all practice models or regions. Similarly, the study relied on self-reporting in the survey: it allows the capture of personal experiences and the impact of WMSDs, but can also be influenced by recall or response biases. Furthermore, as the survey was developed de novo rather than adapted from a single externally validated instrument, the validity of individual items cannot be fully established; it is possible that certain question formats or response-scale structures influenced how participants interpreted and reported their discomfort. Other measures, such as objective ergonomic assessments and clinical examinations, were beyond the scope of this investigation, meaning that specific environmental and postural contributors to discomfort could not be quantified.

These observations highlight several directions for further study. Longitudinal investigation would allow more precise documentation of how WMSD symptoms evolve across career stages and whether targeted ergonomics education produces measurable benefit over time. Future interventional studies incorporating objective measures, such as on-site ergonomic assessments and posture-tracking devices, would also help determine which workplace modifications, equipment changes and scheduling strategies are most effective in reducing musculoskeletal burden in optometric practice.

Overall, this research highlights that WMSDs are common among optometrists, particularly in the neck, shoulders and upper back. Many clinicians manage symptoms independently, and formal ergonomics training or policy support seems limited. These findings suggest that there are meaningful opportunities to strengthen ergonomics education, foster workplace safety changes and develop evidence-based strategies and equipment supporting practitioner comfort and wellbeing.

## Supplementary Information

mskinjuriesOptometry_SurveyQuestions

## Data Availability

Publicly available in a repository: survey data were deposited into the Figshare database and are available at the following 10.6084/m9.figshare.30899024.
