# Supplementary material for: Frequency, Severity and Ergonomic Awareness of Work-Related Musculoskeletal Disorders Among Australian Optometrists: A Cross-Sectional Survey
Source: Ophthalmic Physiol Opt. 2026 Jun 16;46(4):967–74. doi: 10.1007/s44402-026-00118-z (PMC13395821; doi:10.1007/s44402-026-00118-z)
Supplement: Supplementary file 1 — mskinjuriesOptometry_SurveyQuestions [file 44402_2026_118_MOESM1_ESM.docx]

Understanding and mitigating occupational hazards amongst health practitioners

Start of Block: Plain Language Statement and Consent Form

Q1 **Understanding and mitigating occupational hazards amongst health practitioners**

Q2 **Plain Language Statement**
 Department of Optometry and Vision Sciences, The University of Melbourne
 Optometry Australia

**Introduction**
 Thank you for your interest in participating in this research project. The following pages will provide you with further information about the project, so that you can decide if you would like to take part in this research.
 Please take the time to read this information carefully. You may ask questions about anything you don’t understand or want to know more about.
 Your participation is voluntary. If you don’t wish to take part, you don’t have to. If you begin participating, you can also stop at any time.

 **What is this research about?**
 This primary aim of this project is to pinpoint potential causes of occupational injury arising from optometric clinical practices. The secondary aim is to develop tailored solutions aimed at individuals capable of implementing change within the optometric field, fostering a safer and more sustainable working environment for practitioners. This will be achieved via a 30-minute survey targeting optometrists practicing in Australia. The survey aims to gather insights into various facets of occupational injury among optometrists, exploring demographic data, career stages, affected body regions, contextual factors, and employment details. Furthermore, it seeks to delve deeper into the root causes through workplace assessments, focusing on elements such as individuals, tasks, environments, and equipment. Additionally, the survey aims to identify specific trigger scenarios in which these root causes manifest, analysing factors related to movement, practice, environment, and equipment usage within the optometric practice.

 **What are the possible benefits?**
 This survey will serve as an effective tool for identifying specific areas or tasks within the healthcare environment that pose injury risks, allowing for proactive preventive measures. Consequently, addressing these risks and implementing preventive strategies can potentially reduce occupational injury rates among healthcare employees. The data collected through this survey enables informed decision-making, aiding in resource allocation, identifying training needs, or upgrading equipment to mitigate injuries.

 **What will I be asked to do?**
 You will be asked to complete an anonymous online survey that will take 15-30 minutes.

 **What are the possible risks?**
 You may have confidentiality concerns leading you to withhold honest feedback due to fear of repercussions. Additionally, in hierarchical settings, power dynamics might hinder you from expressing concerns about management or working conditions, potentially leading to biased or incomplete survey responses, thereby affecting accuracy. To mitigate these risks, the survey is anonymous and personal information won't be collected to ensure participant data remains confidential. Furthermore, the analysis will focus on a group-level approach to explore the relationship between employees and the network of activity they are involved in, aiming to gather insights without compromising individual privacy. If you need to access support, please contact the project supervisor, Prof. Bang Bui, via email: bvb@unimelb.edu.au or phone: +61 3 8344 7006.

 **Do I have to take part?**
 Participation is voluntary. You may stop at any time. However, once you have submitted survey responses, you will not be able to withdraw because there is no way to identify your anonymous response data.

 **Will I hear about the results of this project?**
 Researchers will analyse the data and write a project report, so you will learn of the outcomes in doing so. Optometry Australia will share the results to its members via newsletter.  Overall, our goal is to publish the complete results of the study in a peer-reviewed journal, where the participants will be anonymised.

 **What will happen to information about me?**
 All data will be stored in a secure way (password secured for digital data) for up to 5 years after the final publication or public release of the study findings and will be destroyed after that. In any academic publication arising from the research, your data will not be individually identifiable (for example, by only presenting average data for the study group, or – if individual data is presented – having individuals referred to by a simple letter code (e.g., Participant A)). With your consent, your anonymous individual digital data may be used in future research related to this project. For example, we may wish to compare your data with similar data we collect in the future.

 **Is there any potential conflict of interest?**
 There is no potential conflict of interest.

 **Who is funding this project?**
 There is no source of external funding. Optometry Australia will provide in-kind administrative support by disseminating the survey and survey results to its members.

 **Where can I get further information?**
 If you would like more information about the project, please contact co-investigator **Prof. Bang Bui** via **email**: bvb@unimelb.edu.au or **phone:** +61 3 8344 7006.

 **Who can I contact if I have any concerns about the project?**
 This project has human research ethics approval from The University of Melbourne [ID 28636]. If you have any concerns or complaints about the conduct of this research project, which you do not wish to discuss with the research team, you should contact the Research Integrity Administrator, Office of Research Ethics and Integrity, University of Melbourne, VIC 3010. tel: (03) 8344 1814 or Email: research-integrity@unimelb.edu.au. All complaints will be treated confidentially. In any correspondence, please provide the name of the research team and/or the name or ethics ID number of the research project.

| Page Break |  |
| --- | --- |

Q3 **Consent Form**
 Department of Optometry and Vision Sciences, The University of Melbourne
 Optometry Australia

 ***Project: Understanding and mitigating occupational hazards amongst health practitioners***

 *Dr Selwyn Prea (Primary Investigator)                                   Email: selwyn.prea@unimelb.edu.au*
 Additional researchers:
 *Prof Bang Bui (Co-Researcher)                                                              Email:* bvb@unimelb.edu.au                                                                                                                     
 *Phone: +61 3 8344 7006*
 *Assoc Prof Kwang Cham (Co-Researcher)                                           Email:* ckwang@unimelb.edu.au
 *Enoch Appathurai ( Co-Researcher)                                                     Email:* abi.a@unimelb.edu.au
 *Nilushi Kodikarage (Doctor of Optometry student researcher)    Email:* n.kodikarage@student.unimelb.edu.au
 *Dylan Loh (Doctor of Optometry student researcher)                     Email:* dylan.loh@student.unimelb.edu.au
 *Duy Ho (Doctor of Optometry student researcher)                          Email:* duy.ho@student.unimelb.edu.au
 *James Burt (Doctor of Optometry student researcher)                   Email:* james.burt@student.unimelb.edu.au
 *Joshua Goundar (Doctor of Optometry student researcher)          Email:* jgoundar@student.unimelb.edu.au

Thank you for your interest in participating in this research project. The following few pages will provide you with further information about the project, so that you can decide if you would like to take part in this research.
 Please take the time to read this information carefully. You may ask questions about anything you don’t understand or want to know more about.
 Your participation is voluntary. If you don’t wish to take part, you don’t have to. If you begin participating, you can also stop at any time.
 By checking the consent box and proceeding to the survey, you are consenting to: Participate in this project, the details of which have been explained to me, and I have been provided with a written plain language statement to keep. I understand that the purpose of this research is to undertake a survey on occupational hazards amongst health practitioners. I understand that my participation in this project is for the purposes outlined in the plain language statement. I acknowledge that the possible effects of participating in this research project have been explained to my satisfaction. In this research I will be invited to participate in an anonymous 20-30 minute survey of work-related stressors, discomfort and body regions, room and equipment design, and treatment seeking behaviour. I understand that my **participation is voluntary**, and any data I contribute is anonymous when data is transcribed and analysed. I understand that I can withdraw any unprocessed data at any time. If I withdraw after submitting my survey responses, it will not be possible to identify my data for withdrawal. I understand that the data from this research will be stored on a password protected University of Melbourne computer and will be destroyed after 5 years. I have been informed that the confidentiality of the information I provide will be safeguarded subject to any legal requirements; my data will be password protected and accessible only by the named researchers. I agree to my data being used as part of a publication on related topics. I understand that my anonymous individual digital data may be used in future research related to this project. I understand that after I respond this consent form will be retained by the researcher. *This research project has been approved by the Human Research Ethics Committee of The University of Melbourne [HREC project ID 28636]. For further information, please contact Prof Bang Bui via* ***email:*** bvb@unimelb.edu.au *or* ***phone:*** *+61 3 8344 7006.*

- I hereby consent to participate as requested in the 'Understanding and mitigating occupational hazards amongst health practitioners' research project (1)
- I do not consent to participate as requested in the 'Understanding and mitigating occupational hazards amongst health practitioners' research project (2)

Skip To: End of Survey If Consent Form Department of Optometry and Vision Sciences, The University of Melbourne Optometry A... = I do not consent to participate as requested in the 'Understanding and mitigating occupational hazards amongst health practitioners' research project

End of Block: Plain Language Statement and Consent Form

Start of Block: Section 1

S1-1 Section 1.

 This section will contain general questions about you and your work as an optometrist.

**Section 1.1 Demographics**

Q1 Gender: How do you identify?

- Male (1)
- Female (2)
- Non-binary / non-conforming (3)
- Prefer not to disclose (4)

Q2 Age: What is your age group (yrs, as at last birthday)?

- 20-29 (1)
- 30-39 (2)
- 40-49 (3)
- 50-59 (4)
- 60-69 (5)
- 70-79 (6)
- Other (7) __________________________________________________

Q3 Height: What is your height (in cm)?

- Please enter here: (1) __________________________________________________
- Prefer not to disclose (2)

Q4 Weight: What is your weight (in kg)?

- Please enter here: (1) __________________________________________________
- Prefer not to disclose (2)

Q5 What is your dominant hand (for work purposes)?

- Left-handed (1)
- Right-handed (2)

Q6 Did you obtain your qualification to practice as an optometrist in Australia or New Zealand?

- Yes (1)
- No (2)

Q7 How many years have you been practicing as an optometrist?

- <5 years (1)
- 5-10 years (2)
- 10-15 years (3)
- >15 years (4)

S1-2 **For the remaining questions on this page, please answer in reference to your work over the last 12 months only**

Q8 On average, how many hours do you work as an optometrist in a typical **WEEK** (include time spent on all tasks such as administration and dispensing)?

- <10 hours (1)
- 10-19 hours (2)
- 20-29 hours (3)
- 30-39 hours (4)
- 40+ hours (5)

Q9 On average, how many patients do you see in a typical **DAY**?

- 0-5 patients (1)
- 6-10 patients (2)
- 11-15 patients (3)
- 16+ patients (4)

| Page Break |  |
| --- | --- |

| 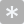 |
| --- |

Q10 Select ALL tasks that you perform at work:

- Eye examinations (1)
- Frame selection (3)
- Dispensing/repairs (5)
- Administration (7)
- Other (please specify) (9) __________________________________________________

Display This Question:

If Select ALL tasks that you perform at work: = Eye examinations

| 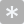 |
| --- |

Q11 How many hours do you spend on eye examinations in a **WEEK**?

________________________________________________________________

Display This Question:

If Select ALL tasks that you perform at work: = Frame selection

| 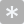 |
| --- |

Q12 How many hours do you spend on frame selection in a **WEEK**?

________________________________________________________________

Display This Question:

If Select ALL tasks that you perform at work: = Dispensing/repairs

| 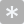 |
| --- |

Q13 How many hours do you spend on dispensing/repairs in a **WEEK**?

________________________________________________________________

Display This Question:

If Select ALL tasks that you perform at work: = Administration

| 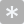 |
| --- |

Q14 How many hours do you spend on administration in a **WEEK**?

________________________________________________________________

| Page Break |  |
| --- | --- |

S1-3 **Section 1.2 Prior understanding of ergonomics and reducing musculoskeletal injuries**
 Posture relates to having your body positioned in a way that makes your work safer, especially when working for long periods or doing repetitive tasks. This means having your head, neck, and torso aligned vertically in a neutral position without pushing your neck forward or tilting your head up or down.

Q15 Are you aware of your posture when practising as an optometrist?

- Always (1)
- Often (2)
- Sometimes (3)
- Rarely (4)
- Never (5)

Q16 How would you rate your posture when practising as an optometrist?

- Excellent (1)
- Above Average (2)
- Average (3)
- Below Average (4)
- Poor (5)

S1-4 Workplace environment

Q17 Does your practice have any Workplace Health and Safety protocols?

- Yes (1)
- No (2)
- Not sure (3)

| 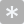 |
| --- |

Q18 When starting at your current workplace, was a risk assessment of your work environment performed?

- Yes (please provide more details) (1) __________________________________________________
- No (2)
- Not sure (3)

| 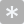 |
| --- |

Q19 When starting at your current workplace, were you provided with any form of training on **reducing musculoskeletal injuries** in your work environment?

- Yes (please provide more details) (1) __________________________________________________
- No (2)
- Not sure (3)

| 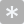 |
| --- |

Q20 When starting at your current workplace, were you provided with any form of training on **setting up and maintaining** a safe and ergonomic work environment?

- Yes (please provide more details) (1) __________________________________________________
- No (2)
- Not sure (3)

S1-5 For the three questions below, please rate how strongly you agree or disagree with the statements provided

Q21 I am aware of the legislations, regulations, and formal guidelines related to maintaining a safe and ergonomic work environment.

- Strongly agree (1)
- Agree (2)
- Neutral (3)
- Disagree (4)
- Strongly disagree (5)

Q22 I have input on the choice of equipment and furnishing in the practice where I work.

- Strongly agree (1)
- Agree (2)
- Neutral (3)
- Disagree (4)
- Strongly disagree (5)

Q23 I have control over my pace of work e.g., length of appointments, appointment scheduling, lunch and other comfort breaks.

- Strongly agree (1)
- Agree (2)
- Neutral (3)
- Disagree (4)
- Strongly disagree (5)

S1-6 Education and Continuing Professional Development

| 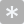 |
| --- |

Q24 As part of obtaining your university qualification, were you provided with any form of training on **reducing musculoskeletal injuries** in your work environment?

- Yes (please provide more details) (1) __________________________________________________
- No (2)
- Not sure (3)

| 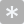 |
| --- |

Q25 As part of obtaining your university qualification, were you provided with any form of training on setting up and maintaining a **safe and ergonomic work environment**?

- Yes (please provide more details) (1) __________________________________________________
- No (2)
- Not sure (3)

| 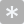 |
| --- |

Q26 Have you attended any continuing professional development (CPD) activity specifically related to **ergonomics in the optometry profession**?

- Yes (please provide more details) (1) __________________________________________________
- No (2)
- Not sure (3)

S1-7 Personal factors

| 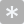 |
| --- |

Q27 Have you implemented any strategies to reduce musculoskeletal injuries in the work environment?

- Yes (please provide more detail) (1) __________________________________________________
- No (2)

Q28 What are some of the challenges/barriers regarding reducing musculoskeletal injuries in your current work environment?

________________________________________________________________

________________________________________________________________

________________________________________________________________

________________________________________________________________

________________________________________________________________

End of Block: Section 1

Start of Block: Section 2

S2-1 **Section 2.**
 Please indicate whether you have experienced any of the following musculoskeletal discomfort **in the past 12 months** whilst practicing as an **optometrist**.

 The term “discomfort” includes pain, ache, difficulty with movement and numbness. In this section, please only respond to work-related discomfort.


 Figure 1 Diagram of body with approximate areas of pain labelled

 In this picture you can see the approximate position of the parts of the body referred to in this survey. Limits are not sharply defined, and certain parts overlap. You should decide for yourself in which part you have or have had your trouble (if any). Adapted from Kuorinka et. al., 1987.

| 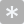 |
| --- |

Q29 In the past 12 months, have you experienced any…

|  | Yes (1) | No (2) |
| --- | --- | --- |
| Neck discomfort (1) |  |  |
| Shoulder discomfort (2) |  |  |
| Upper back discomfort (3) |  |  |
| Lower back discomfort (4) |  |  |
| Elbow discomfort (5) |  |  |
| Wrist/hand discomfort (6) |  |  |
| Knee discomfort (7) |  |  |
| Ankle/foot discomfort (8) |  |  |

Carry Forward Selected Choices from "In the past 12 months, have you experienced any…"

| 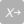 |
| --- |

Q30 Please rate the severity of the symptoms on a scale from 0 to 10 (0 being no discomfort and 10 being severe discomfort)

|  | 0 | 1 | 2 | 3 | 4 | 5 | 6 | 7 | 8 | 9 | 10 |
| --- | --- | --- | --- | --- | --- | --- | --- | --- | --- | --- | --- |

| Neck discomfort () | 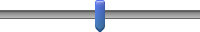 |
| --- | --- |
| Shoulder discomfort () | 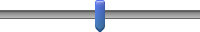 |
| Upper back discomfort () | 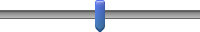 |
| Lower back discomfort () | 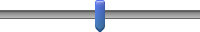 |
| Elbow discomfort () | 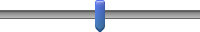 |
| Wrist/hand discomfort () | 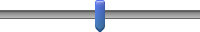 |
| Knee discomfort () | 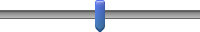 |
| Ankle/foot discomfort () | 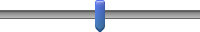 |

S2-2 The following pages will ask for more specific information about each area of discomfort that you have indicated experiencing. You only need to provide information in each section that applies to the specific discomfort you have indicated. For example, if you only experience neck discomfort, answer this section in relation to your neck discomfort only. If you experience discomfort in more than one region, you will complete the questionnaire for each body region.

End of Block: Section 2

Start of Block: Section 3

S2-1-1 **Section 3.** *(Repeated for each of the areas in which discomfort has been experienced)*
 Duration of Discomfort and Contributing Factors

Q31 At any point during the **last 7 days**, have you experienced work-related ${lm://Field/2} discomfort?

- Yes (1)
- No (2)

Q32 In the **last 12 months**, in what situation was the work-related ${lm://Field/2} discomfort experienced?

- Only while working (1)
- While working and several hours after ceasing work (2)
- Present all the time (3)

Q33 In the **last 12 months**, what is the total cumulative time length that you have experienced work-related ${lm://Field/2} discomfort?

- Less than 1 day (1)
- 1-7 days (2)
- 8-30 days (3)
- More than 30 days, but not everyday (4)
- Everyday (5)

Q34 When did the work-related ${lm://Field/2} discomfort start?

- Less than 3 months (1)
- Between 3 months to 1 year (2)
- Between 1 year to 5 years (3)
- Over 5 years (4)

S2-1-2 Contributing factors The following list describes tasks at work that could contribute to work-related discomfort.

| 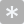 |
| --- |

Q35 Which of the following factors contributed to the work-related ${lm://Field/2} discomfort?
Select **ALL** that apply:

- Performing repetitive tasks (1)
- Examining a large number of patients per day (2)
- Insufficient rest breaks during the workday (3)
- Working in awkward and cramped positions (4)
- Working in the same position for long periods (e.g. standing, bending over, sitting) (5)
- Bending or twisting your back in an awkward way (6)
- Working near or at your physical limits (7)
- Reaching or working away from your body (8)
- Continuing to work while experiencing discomfort, injured or hurt (9)
- Lifting or moving dependant patients (10)
- Carrying, lifting or moving heavy objects or equipment (11)
- Work scheduling (overtime, length of workday) (12)
- Other factors (please provide more details) (13) __________________________________________________

| 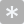 |
| --- |

Q36 Which of the following procedures increases your work-related ${lm://Field/2} discomfort?
Select ALL that apply:

- Phoropter (1)
- Slitlamp/fundoscopy/gonioscopy (2)
- Ophthalmoscopy (3)
- Computer and writing tasks (4)
- Trial frame / trial lenses / hand held equipment, for example, occluder, prism bars (5)
- Other (please provide more details) (6) __________________________________________________

| 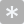 |
| --- |

Q37 Have you modified your work and/or your workspace to address the work-related ${lm://Field/2} discomfort?

- Yes (please see next question) (1)
- ⊗No (please explain any reasons why not) (2) __________________________________________________

Display This Question:

If Have you modified your work and/or your workspace to address the work-related ${lm://Field/2} dis... = Yes (please see next question)

| 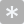 |
| --- |

Q37-b If yes, please select the modifications you have implemented

- Adjust posture (1)
- Adjust equipment (2)
- Perform alternative procedures (3)
- Alter work schedule (4)
- Stretching and relaxation exercises (5)
- Other (please provide more details) (6) __________________________________________________

Display This Question:

If Loop current: Have you modified your work and/or your workspace to address the work-related ${lm://Field/2} dis... = Yes (please see next question)

| 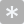 |
| --- |

Q37-c Were the modifications implemented effective in addressing the work-related ${lm://Field/2} discomfort?

|  | Was it effective? | | Why/Why not? |
| --- | --- | --- | --- |
|  | Yes (1) | No (2) | Please provide more information (1) |
| Adjust posture (1) |  |  |  |
| Adjust equipment (2) |  |  |  |
| Perform alternative procedures (3) |  |  |  |
| Alter work schedule (4) |  |  |  |
| Stretching and relaxation exercises (5) |  |  |  |
| Other modifications (6) |  |  |  |

S2-1-3 Impact Health

| 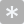 |
| --- |

Q38 Have you seen a healthcare professional because of work-related ${lm://Field/2} discomfort?

- Yes, a medical practitioner (eg. general practitioner (GP) or a specialist ) (1)
- Yes, an allied health practitioner (eg. physiotherapy, occupational therapy, or chiropractic care) (2)
- Yes, an alternative therapy provider (eg. naturopathy, acupuncture, or remedial massage) (3)
- ⊗No (please explain any reasons why not) (4) __________________________________________________

Display This Question:

If Loop current: Have you seen a healthcare professional because of work-related ${lm://Field/2} discomfort? != No (please explain any reasons why not)

And And Have you seen a healthcare professional because of work-related ${lm://Field/2} discomfort? q://QID340/SelectedChoicesCount Is Greater Than or Equal to 1

Q38-b Were the modifications implemented effective in addressing the work-related ${lm://Field/2} discomfort?

|  | Was it effective? | | Why/Why not? |
| --- | --- | --- | --- |
|  | Yes (1) | No (2) | Please provide more information (1) |
| A medical practitioner (1) |  |  |  |
| An allied health practitioner (2) |  |  |  |
| An alternative therapy provider (3) |  |  |  |

| 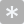 |
| --- |

Q39 Have you taken medication to address the work-related ${lm://Field/2} discomfort?

- Yes (1)
- No (please explain any reasons why not) (2) __________________________________________________

Display This Question:

If Loop current: Have you taken medication to address the work-related ${lm://Field/2} discomfort? = Yes

| 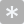 |
| --- |

Q39-b Was medication effective in addressing the work-related ${lm://Field/2} discomfort?

- Yes (please provide a reason for your answer) (1) __________________________________________________
- No (please provide a reason for your answer) (2) __________________________________________________

Q40 Have you ever been hospitalised because of the work-related ${lm://Field/2} discomfort?

- No (1)
- Yes, for less than a day (2)
- Yes, overnight (3)
- Yes, for a week or less (4)
- Yes, for a month or less (5)
- Yes, for longer than a month (6)

S2-1-4 Daily living

Q41 Have you had to change jobs because of the work-related ${lm://Field/2} discomfort?

- Yes (1)
- No (2)

| 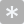 |
| --- |

Q42 Has the work-related ${lm://Field/2} discomfort prevented you from performing certain tasks?

|  | Yes (1) | No (2) |
| --- | --- | --- |
| Work-related activities (1) |  |  |
| Home or leisure activities (2) |  |  |

Display This Question:

If Has the work-related ${lm://Field/2} discomfort prevented you from performing certain tasks? = Work-related activities [ Yes ]

Q42-b-1 In the **last 12 months**, what is the approximate duration that work-related ${lm://Field/2} discomfort has prevented you from doing tasks at work?

- 0 days (1)
- 1-7 days (2)
- 8-30 days (3)
- More than 30 days (4)

Display This Question:

If Has the work-related ${lm://Field/2} discomfort prevented you from performing certain tasks? = Home or leisure activities [ Yes ]

Q42-b-2 In the **last 12 months**, what is the approximate duration that work-related ${lm://Field/2} discomfort has prevented you from doing tasks at home?

- 0 days (1)
- 1-7 days (2)
- 8-30 days (3)
- More than 30 days (4)

End of Block: Section 3

Start of Block: Section 4

Q43 **Section 4.**

 Final Comments

Q44 Do you have any other comments relating to musculoskeletal injuries and discomfort in the practice of optometry?

________________________________________________________________

________________________________________________________________

________________________________________________________________

________________________________________________________________

________________________________________________________________

End of Block: Section 4
